# Automated minute scale RNA-seq of pluripotent stem cell differentiation reveals early divergence of human and mouse gene expression kinetics

**DOI:** 10.1371/journal.pcbi.1007543

**Published:** 2019-12-09

**Authors:** Christopher Barry, Matthew T. Schmitz, Cara Argus, Jennifer M. Bolin, Mitchell D. Probasco, Ning Leng, Bret M. Duffin, John Steill, Scott Swanson, Brian E. McIntosh, Ron Stewart, Christina Kendziorski, James A. Thomson, Rhonda Bacher

**Affiliations:** 1 Morgridge Institute for Research, Madison, WI, United States of America; 2 Department of Biostatistics and Medical Informatics, University of Wisconsin-Madison, Madison, WI, United States of America; 3 Department of Cell and Regenerative Biology, University of Wisconsin School of Medicine and Public Health, Madison, WI, United States of America; 4 Department of Molecular, Cellular, and Developmental Biology, University of California, Santa Barbara, CA, United States of America; 5 Department of Biostatistics, University of Florida, Gainesville, FL, United States of America; EMBL-Heidelberg, GERMANY

## Abstract

Pluripotent stem cells retain the developmental timing of their species of origin *in vitro*, an observation that suggests the existence of a cell-intrinsic developmental clock, yet the nature and machinery of the clock remain a mystery. We hypothesize that one possible component may lie in species-specific differences in the kinetics of transcriptional responses to differentiation signals. Using a liquid-handling robot, mouse and human pluripotent stem cells were exposed to identical neural differentiation conditions and sampled for RNA-sequencing at high frequency, every 4 or 10 minutes, for the first 10 hours of differentiation to test for differences in transcriptomic response rates. The majority of initial transcriptional responses occurred within a rapid window in the first minutes of differentiation for both human and mouse stem cells. Despite similarly early onsets of gene expression changes, we observed shortened and condensed gene expression patterns in mouse pluripotent stem cells compared to protracted trends in human pluripotent stem cells. Moreover, the speed at which individual genes were upregulated, as measured by the slopes of gene expression changes over time, was significantly faster in mouse compared to human cells. These results suggest that downstream transcriptomic response kinetics to signaling cues are faster in mouse versus human cells, and may offer a partial account for the vast differences in developmental rates across species.

## Author summary

For regenerative medicine, a slow developmental clock operating in human pluripotent stem cells imposes severe obstacles to many pluripotent stem cell therapies. Producing the desired cell types with the necessary level of physiological maturity in clinically-relevant time frames will be essential if the therapy is aimed at being patient-specific. Thus, understanding what controls species-specific rates of differentiation and identifying ways to accelerate it would have a broad impact on improving the therapeutic value of human pluripotent stem cells. In this paper, we measured the immediate-early gene expression responses to neural differentiation signals in mouse and human pluripotent stem cells. We quantitatively summarized the gene expression patterns using a flexible model that allowed us to identify shared trends and dynamic changes across species. We found that responses occurred equally early across species, but expression patterns were prolonged in human cells and the rate of upregulation in gene expression was significantly faster in mouse cells. Our results represent a significant contribution in furthering the knowledge of early developmental transcriptional programs.

## Introduction

Pluripotent stem cells, due to their properties of self-renewal and potency, provide a unique model for studying early development and are of particular interest for their exciting potential in regenerative therapies [1]. In order to understand the behavior and timing of pluripotent stem cell differentiation, both human and mouse models have been studied and compared. Although it is widely accepted that mouse pluripotent stem cells differentiate more quickly than human cells [2–6], the underlying causes for species-specific differences in developmental rates are still unknown.

Indeed, human and mouse development occur on vastly different time scales. Humans develop over an approximately nine-month gestation, whereas mice require approximately three weeks. Between animals of different sizes, the interval between conserved developmental milestones scales with body size, allowing additional growth between key transitions [7,8]. For example, the time required to progress from neural plate formation to neural tube closure is stretched in the human compared to the mouse embryo. The neural plate forms at embryonic day 7.5 in the mouse and requires only 2 additional days to fully close the neural tube in the embryo, growing roughly 2.6 mm in that short time [9]. In contrast, the neural plate in human embryos begins to form around embryonic day 18 and requires 10 days for neural tube closure, wherein the embryo grows roughly 2.8 mm [10]. Within each species, growth and differentiation rates are tightly coordinated during embryonic growth. Remarkably, it has also been shown that pluripotent cells closely maintain their species-specific developmental rates *ex utero*, even in the absence of an intact embryo or the maternal environment [2–5,11–14]. Together, this suggests the existence of a species-specific cell-intrinsic clock contributing to developmental timing, although the mechanisms orchestrating developmental timing across vast differences in body size remain largely unknown [14,15].

During embryonic development and *in vitro* stem cell differentiation alike, signaling factors bind receptors on the plasma membrane to initiate complex signal transduction cascades that ultimately result in the regulation of genes that drive lineage commitment programs [16,17]. We therefore hypothesized that the rate at which extracellular signaling factors induce changes in gene expression is different across species. Any observed differences in expression could be affected by two possible models: (1) the time required for extracellular signaling to reach the nucleus could be different across species, or (2) the transcriptional response kinetics in the nucleus could operate at different speeds across species.

To explore this question, here we used a custom automated liquid-handling cell culture robotic system to collect RNA-sequencing (RNA-seq) data at a scale of minutes during the initiation of neural differentiation of human and mouse pluripotent stem cells. We report surprisingly early, rapid, and dynamic gene expression patterns in both human and mouse stem cells within minutes of differentiation, identifying biological processes that would otherwise be concealed in daily or even hourly time courses. We find evidence that innate species-specific differences in transcriptional response rates to differentiation factors may therefore explain, at least in part, the differences in developmental rates observed across species.

## Results

**Fig 1:**
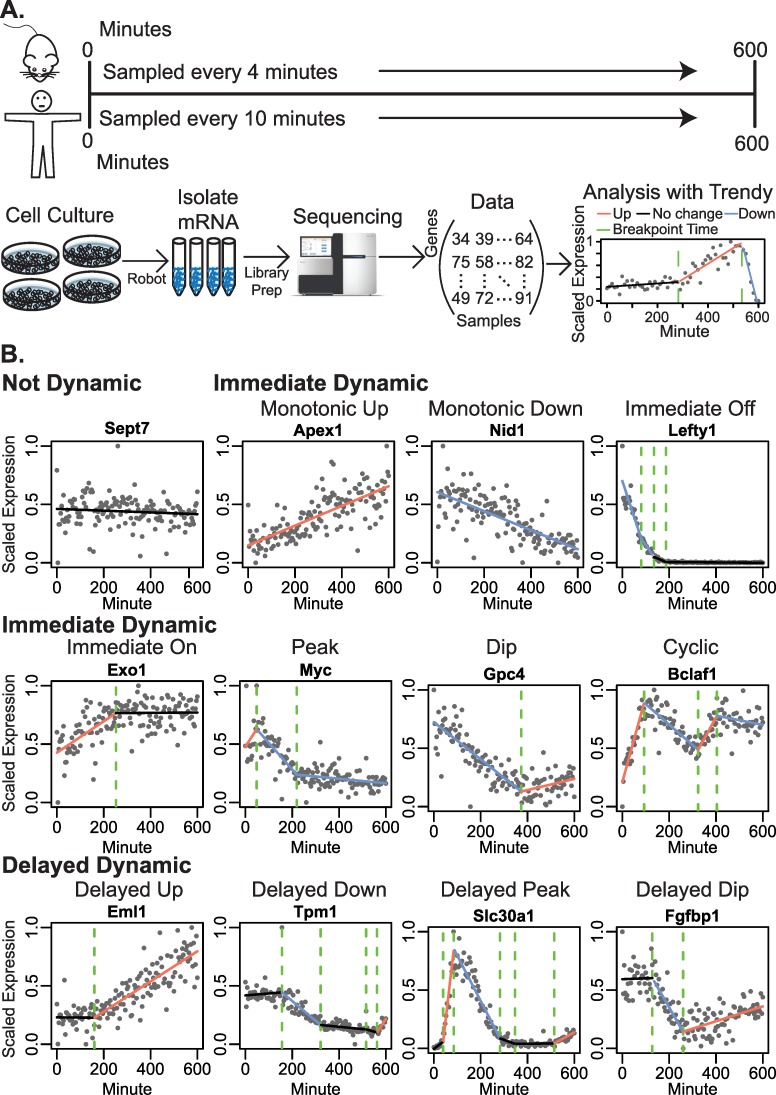
Experiment overview and expression patterns. A) Mouse and human pluripotent stems cells were exposed to neural differentiation factors to initiate differentiation and then sampled for RNA-sequencing every 4 or 10 minutes for the first 600 minutes. Expression trends along the time course for each gene were fit using a breakpoint model. B) Expression trends are shown for representative genes having patterns classified as not dynamic, immediate dynamic, or delayed dynamic. Immediate dynamic genes had an immediate up- or downregulation segment, whereas delayed dynamic genes had a flat initial segment. Trends were further characterized as having a peak, dip, or as cyclic (multiple peak/dip) (Methods).

We set out to directly compare initial response rates of mouse and human pluripotent stem cell differentiation by exposing them to identical neural differentiation conditions and monitoring changes in gene expression at high temporal frequency. Human embryonic stem (hES) cells and mouse epiblast stem (mEpiS) cells are widely considered to represent primed (post-implantation) stages of embryonic development [18–21] and were therefore used as comparable developmental starting points. Prior to differentiation, hES cells or mEpiS cells were seeded in multi-well robot culture plates under pluripotent growth conditions. All cell seeding, media changes, washes, and lyses were performed by a TECAN Freedom Evo200 liquid handling robot in a controlled environmental enclosure. At the onset of differentiation (time 0), the media was replaced with neural induction media pre-warmed to 37°C. Mouse cells were lysed every 4 minutes (+/- 2 seconds), the highest sampling frequency of our robotic system given the number of plates, for the first 600 minutes of neural induction, and human cells were lysed at 10-minute (+/- 2 seconds) intervals in order to maximize the efficiency for sample numbers and sequencing costs while still achieving the sampling frequency necessary to capture the 2:5 ratio of differentiation speeds observed *in utero* and *in vitro* [14] (Fig 1A). This collection resulted in a total of 145 RNA-seq time-points for mouse and 61 time-points for human after quality control (Methods).

Dynamic gene expression patterns over the time course in the mouse and human datasets were identified by fitting a set of segmented regression models to the expression of each gene using the R package Trendy [22] (Fig 1A). A segmented regression model represents each gene’s expression as a linear piece-wise function over time, with each segment composed of consecutive time-points. Adjoining connecting segments are breakpoints that identify discrete points of change in expression (Fig 1A). For each gene, we estimated the number and location of breakpoints, allowing a maximum of five possible breakpoints or changes in expression across the entire time course and only retaining the best fit model (Methods). The large number of time-points generated from high frequency sampling enables the detection of many possible expression patterns. The segmented model is therefore flexible and not restricted to pre-defined expression trends (Section 1 in S1 Text). The best fit model reports parameters on the time of breakpoints and the direction and slope of each segment. We refer here to genes according to the defined patterns shown in Fig 1B. For example, genes that displayed a single peak profile were those having an up segment followed by a down segment. All genes which had an initial up- or down- trend are referred to as immediate dynamic genes, while those with an initially flat trend are referred to as delayed dynamic genes (Fig 1B and Methods). An overview of the number of genes for each pattern is given in S1 Table.

### Changes in gene expression patterns occur earlier in mouse versus human pluripotent stem cells despite similarly early onset of responses

To determine if patterns in gene expression changed at different rates across species, we first examined gene orthologs with dynamic trends in both human and mouse cells. We used the adjusted coefficient of determination statistic (R^2^) as a goodness of fit measure for the segmented regression model to eliminate genes not having a dynamic trend or those with noisy expression. There were 1436 genes having an adjusted R^2^ > 0.2 on their fitted gene expression trends in both human and mouse time courses (S2 Table and Section 2 in S1 Text; S3 Table). Consistent with capturing events related to early development, many of these orthologs were identified as binding targets for transcription factors related to differentiation including *SP1*, *SMAD4*, and *SOX2* as well as cell cycle-related genes *TP53* and *E2F1* (S4 Table).

**Fig 2:**
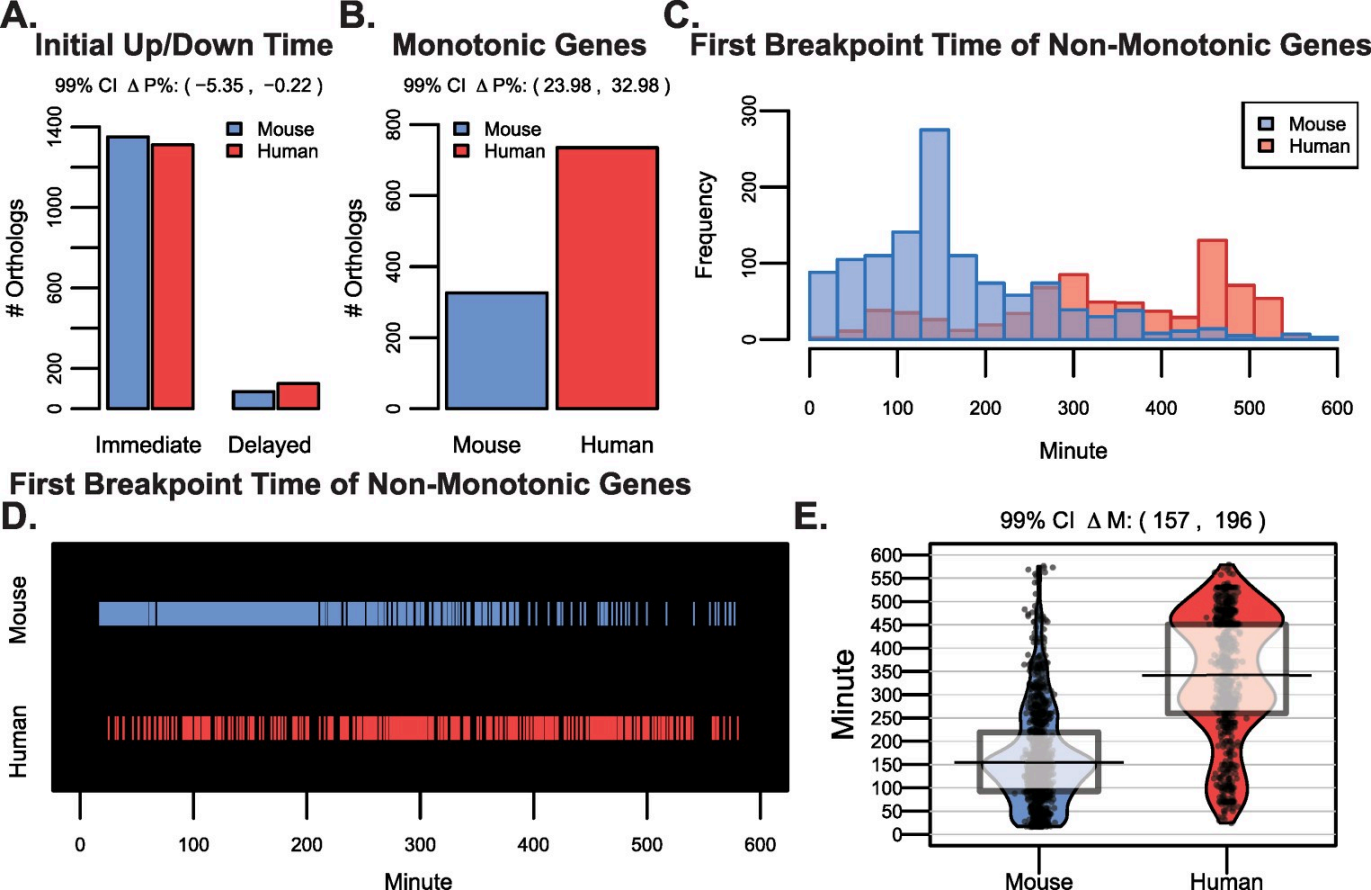
Initial human and mouse ortholog expression trends begin at similar times yet last for longer in human. A) The number of genes having an up or down trend occurring immediately versus those displaying a delayed trend is shown. The two mouse/human bars represent genes having either immediate or delayed dynamics. The 99% confidence interval for the difference in the percent (ΔP%) of orthologs having an immediate up/down trend was -5.35% to -0.22%. B) The number of orthologs that had monotonic expression patterns (i.e. no breakpoints) along the time courses. The 99% confidence interval for the difference in the percent (ΔP%) of monotonic orthologs in human versus mouse was 23.98% to 32.98%. C) For non-monotonic genes (i.e. those having at least one breakpoint), histograms of the first breakpoint time are shown for mouse and human genes. D) Similar to C), the time of the first breakpoint is highlighted in either blue or red for mouse and human orthologs. E) The first breakpoint times for mouse and human orthologs are represented as smoothed densities (bean plots) with overlaid raw data, the median, and a box representing the interquartile range. The 99% confidence interval on the paired Wilcoxon rank sum statistic for shift in distribution (ΔM) was 157 to 196 minutes.

We compared the percentage of orthologous genes having an immediate dynamic expression trend to assess whether there were species-specific differences in the time of initial up- or downregulation in response to differentiation cues. Any initial increase or decrease in expression that occurred within the first 20 minutes in mouse cells or 30 minutes in human cells was considered as being ‘immediately’ dynamic (Methods and Sections 3 & 4 in S1 Text). Surprisingly, the vast majority of orthologs that displayed dynamic expression trends for both mouse and human genes did so immediately (Fig 2A). 91% of dynamic orthologs were immediately up- or downregulated upon initiation of human cell differentiation and had the initial up or down trend last for at least 30 minutes, and 94% were similarly immediately regulated in mouse cells for at least 20 minutes. Despite no difference between species initial onset of gene expression changes, we did observe differences in the lengths of those up- and downregulated segments. There were 2.3 times as many orthologs that continued trending up or down in expression undisturbed (monotonic expression) in human (735 orthologs) compared to mouse cells (326 orthologs) (Fig 2B). For non-monotonic genes, we next examined how long the initial trend lasted. The first breakpoint estimated by the segmented regression model represents the time of the expression change following the initial response. Orthologs had an overwhelmingly earlier first breakpoint time in mouse compared to human cells (Fig 2C–2E). The median shift in mouse breakpoints was 177 minutes earlier than for human genes (99% confidence interval [CI] 157 to 196 minutes) with 80.3% of first breakpoints occurring within the first 250 minutes of mouse differentiation compared to only 23.1% of human ortholog breakpoints.

**Fig 3:**
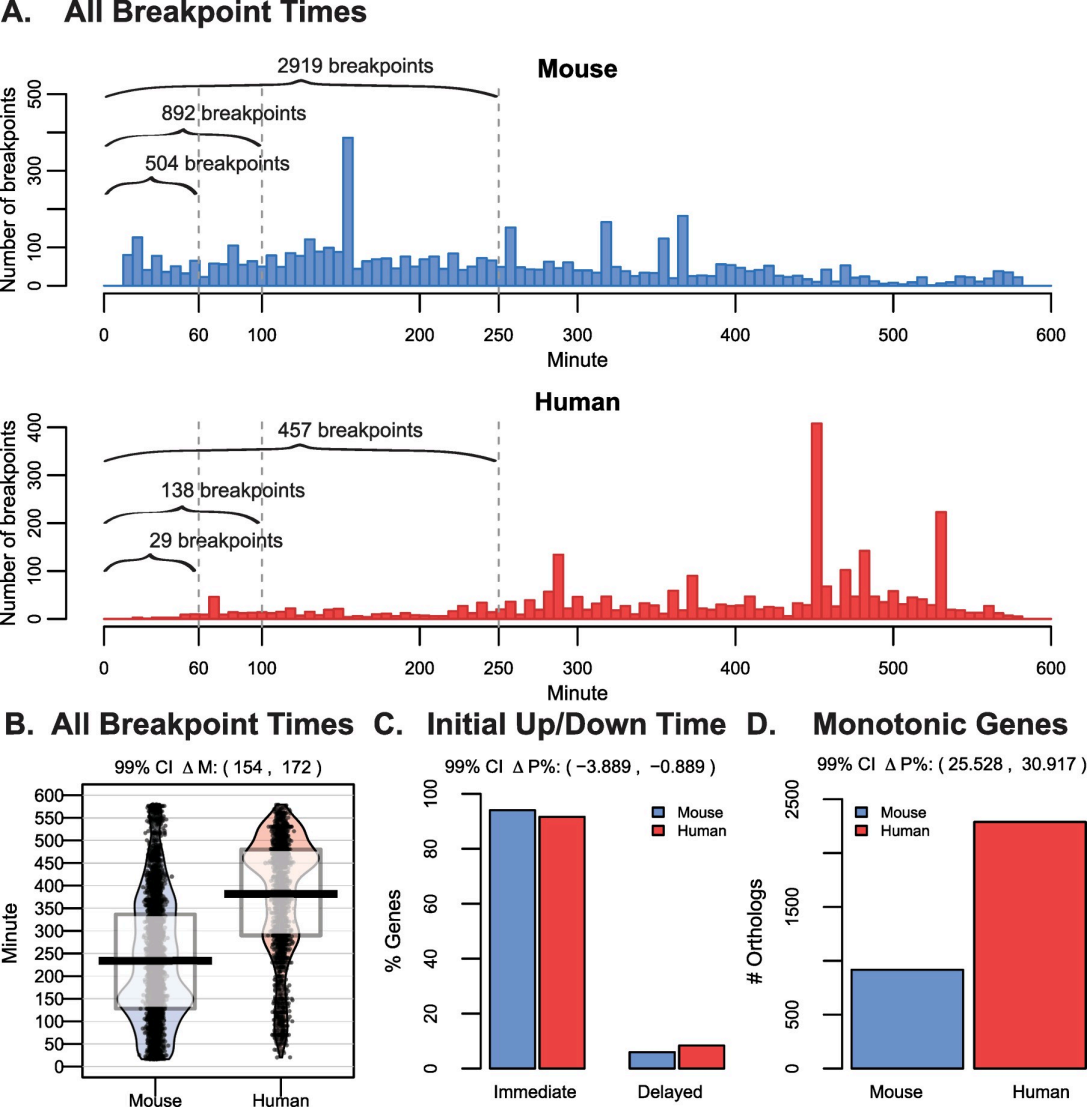
Dynamic genes in either mouse or human experiments also display earlier breakpoints in mouse compared to human cells. (A) The distribution of all breakpoint times among 3721 dynamic genes in Mouse and 4332 dynamic genes in Human. The values indicate the number of breakpoints occurring prior to 60 minutes, 100 minutes, and 250 minutes. Some genes may have multiple breakpoints in each time-frame. B) Smoothed density plot as previously described are shown for all breakpoint times among mouse and human dynamic genes. The 99% confidence interval for the Wilcoxon rank sum statistic shift in distribution (ΔM) for breakpoint times in human versus mouse was 154 to 172 minutes. C) The percent of genes having an up or down trend immediately versus those displaying a delayed trend (a period of stable expression for five consecutive time-points in mouse cells or three consecutive time-points in human cells followed by an up or down response). The 99% confidence interval for the difference in the percent (ΔP%) of genes having an immediate up/down trend was -3.89% to -0.89%. D) The number of genes that had monotonic expression patterns (i.e. no breakpoints) along the time course trending either up or down. The 99% confidence interval for the difference in the percent (ΔP%) of monotonic genes was 25.5% to 30.9%.

The results above held when we expanded the analysis to *all* genes in *either* species with an adjusted R^2^ > 0.2, broadening the scope to 3721 dynamic mouse genes and 4332 dynamic human genes identified by segmented regression analysis. Within the first 60 minutes, there were 504 breakpoints or changes in gene expression among 497 genes in mouse cells, meaning a handful of genes had multiple changes in expression in this time, while only 29 genes had a breakpoint before the first hour in human cells (Fig 3A). At 100 minutes post-differentiation, there were 892 total breakpoints in mouse cells, yet only 138 in human cells, and after the first 250 minutes, there were 2919 breakpoints in mouse cells and 457 breakpoints in human cells (Fig 3A). Overall, the median breakpoint time was significantly earlier in mouse compared to human genes (Fig 3B; 99% CI on ΔM 154 to 172 minutes). For both species, over 90% of all dynamic genes were up- or downregulated immediately within 20 minutes of post-differentiation for mouse and 30 minutes for human (Fig 3C). When considering all dynamic genes, again human cells had 2.5 times more monotonic gene expression compared to mouse cells (2289 in human versus 916 in mouse) (Fig 3D).

We additionally confirmed that the difference in breakpoint times was not related to differences in sampling frequencies between the experiments. We reran the analysis above on a subset of the mouse time course such that sampling rate was effectively reduced to only every 12 minutes (every 3^rd^ sample) (Methods). We found that mouse cells retained their earlier breaktimes and had fewer monotonic genes compared to human cells (Figure A-C in S1 Fig), consistent with our analysis of the entire data set.

We next wanted to confirm that the divergences in gene expression kinetics we observed were specific to developmental events rather than other cellular events affected by the addition of fresh medium. We repeated the time course experiment in human stem cells but added pluripotency medium to cells rather than neural differentiation medium as a control for tissue culture effects unrelated to differentiation.

We rationalized that human cells would provide the most stringent test as they would be more likely to exhibit similar changes in gene expression across conditions given their less rapid and dynamic responses compared to mouse cells. Highly dynamic gene responses, such as those in mouse cells, might overestimate the differences between the two conditions and mask similarities induced by feeding effects alone.

Of the 4332 dynamic genes identified by our segmented regression analysis during the initiation of human stem cell differentiation, only 1634 (37.6%) of them also had dynamic trends during pluripotent medium feeding (S2 Fig). Furthermore, of those 1634 genes, the majority (59% of the shared dynamic genes, or 22.1% of all dynamic genes in the control experiment) were regulated differently, having initial trends in the opposite direction, between the differentiation and the maintenance of pluripotency time courses (Figure A in S2 Fig). The distribution of breakpoint times between the experiments is also quite different with the control time course displaying systematic breakpoint activity (Figure B in S2 Fig). The systematic global break times in the feeding control time course were enriched for cell cycle and WNT signaling pathway genes, consistent with the conservation of pluripotency and propagation of cell proliferation, while the major break times in the differentiation time course were enriched in neuron differentiation and cell development-related genes (Figure C in S2 Fig & S5 Table). All together, these results suggest that the gene expression effects detected in our cross-species differentiation time courses were responses intrinsic to stem cell differentiation rather than artifacts of cell feeding.

### Peaks in gene expression occur earlier in mouse vs human cells during the initiation of *in vitro* differentiation

**Fig 4:**
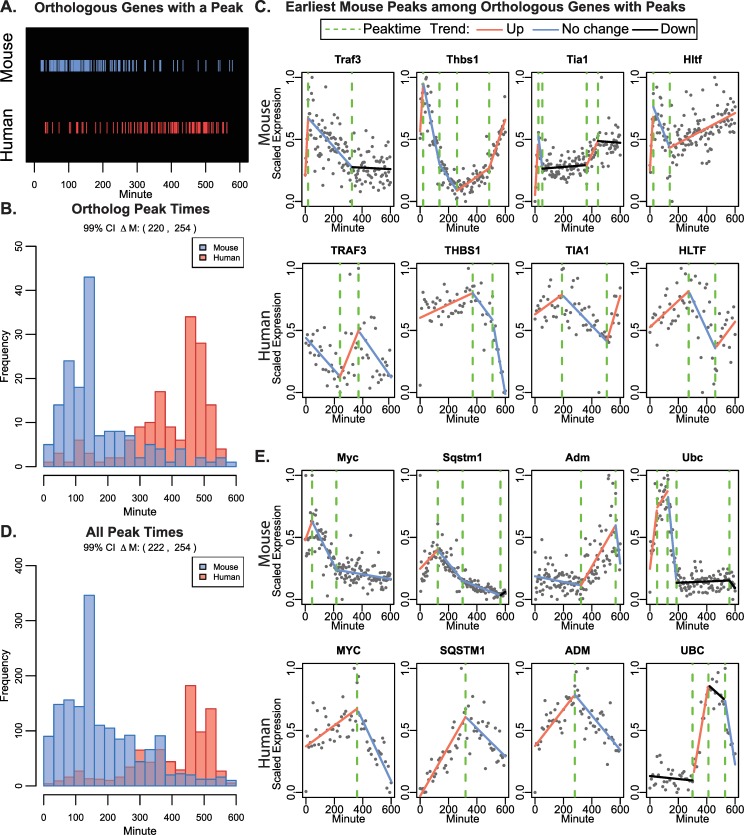
Gene peaks occur earlier in mouse compared to human cells. (A) The sorted peak time of ortholog genes highlighted in either blue or red for mouse and human orthologs, respectively. B) Histogram of the peak time for orthologs. The 99% confidence interval on the paired Wilcoxon rank sum statistic for shift in distribution (ΔM) was 220 to 254 minutes. C) Scatter plots of scaled normalized expression for the first four peaking orthologs (first in mouse) are shown with the breakpoint model fit from Trendy overlaid. The peak time is shown as a green dotted line. D) Histogram of the peak time for genes having a peak in either species. The 99% confidence interval on the Wilcoxon rank sum statistic for shift in distribution (ΔM) was 222 to 254 minutes. E) Scatter plots of scaled normalized expression for four peaking, immediate-early orthologs are shown with the breakpoint model fit overlaid and peak times indicated by the green dotted lines.

The most common pattern among the set of 1436 dynamic ortholog genes was the peak trend (including both immediate and delayed) with 156 orthologs having a peak in both mouse and human cells (S6 Table). Here we consider genes that have any peak along the time course, including cyclic genes (Methods). The median time of the peak for mouse ortholog genes occurred 238 minutes earlier compared to their peak time in human cells (Fig 4A and 4B; 99% CI on ΔM 220 to 254 minutes). When comparing the plots of the first four common peaks individually, the genes display striking differences in the species-specific timing of their peaks (Fig 4C). We did not find any overall correlation between the mouse and human peak times (S3 Fig), though among the 156 peak orthologs there was significant enrichment of GO-terms related to proliferation, differentiation, and neurogenesis (S7 Table). When we expanded our analysis to include the entire transcriptome with significantly fitted gene expression patterns in either mouse or human cells over the 10-hour time course, our observations again held true as we found that mouse genes peaked significantly earlier (99% CI on ΔM 222 to 254 minutes), with 1589 mouse genes having a peak compared to 840 human genes (Fig 4D; S8 Table).

Previous reports indicate that gene peak patterns tend to be enriched in genes identified as ‘immediate-early response’ genes (IEG) [23]. IEGs can exhibit immediate transcriptional responses to stimuli and generally have easily accessible promoters which allow them to be regulated more quickly by transcription factors [24]. Many IEGs themselves encode for transcription factors [24]. Thirteen of the peaking orthologs we identified in our time course analysis are classified as immediate early genes among a literature-curated list from Arner et al., 2015 [25], with only 51 out of 1414 orthologs overall having this classification. Of the 13 IEG peak orthologs *Myc*, *Jun*, *Btg2* and *Atf4* are transcription factors, although the others such as *Sqstm1*, *Adm*, and *Ubc* are all genes known have protein products involved in neuronal differentiation [26–28] (Fig 4E). Since both the human and mouse cells received identical neural induction media, any differences in the immediate early response genes represent species-specific differences in differentiation. We compared the differences in peak time among the IEGs (S1 File), and found 9 of 13 had immediate upregulation, 3 had delayed upregulation in human cells and 1 had immediate down regulation in mouse. When comparing to the larger set of all peaks within each species, there were 39 and 34 peaking immediate-early genes in the mouse and human time courses, respectively, indicating that peaking genes are enriched with previously known IEGs and represent developmentally relevant trends.

### Divergence in the rate of change in gene expression across species

We wanted to assess which components of the gene expression patterns led to the differences in timing we observed. There are two non-exclusive explanations for the earlier peak times observed in mouse compared to human cells: (1) the “up” segment leading towards the peak begins earlier in mouse compared to humans, and/or (2) the slope of the segment leading towards the peak, or rate of change in gene expression, is steeper.

**Fig 5:**
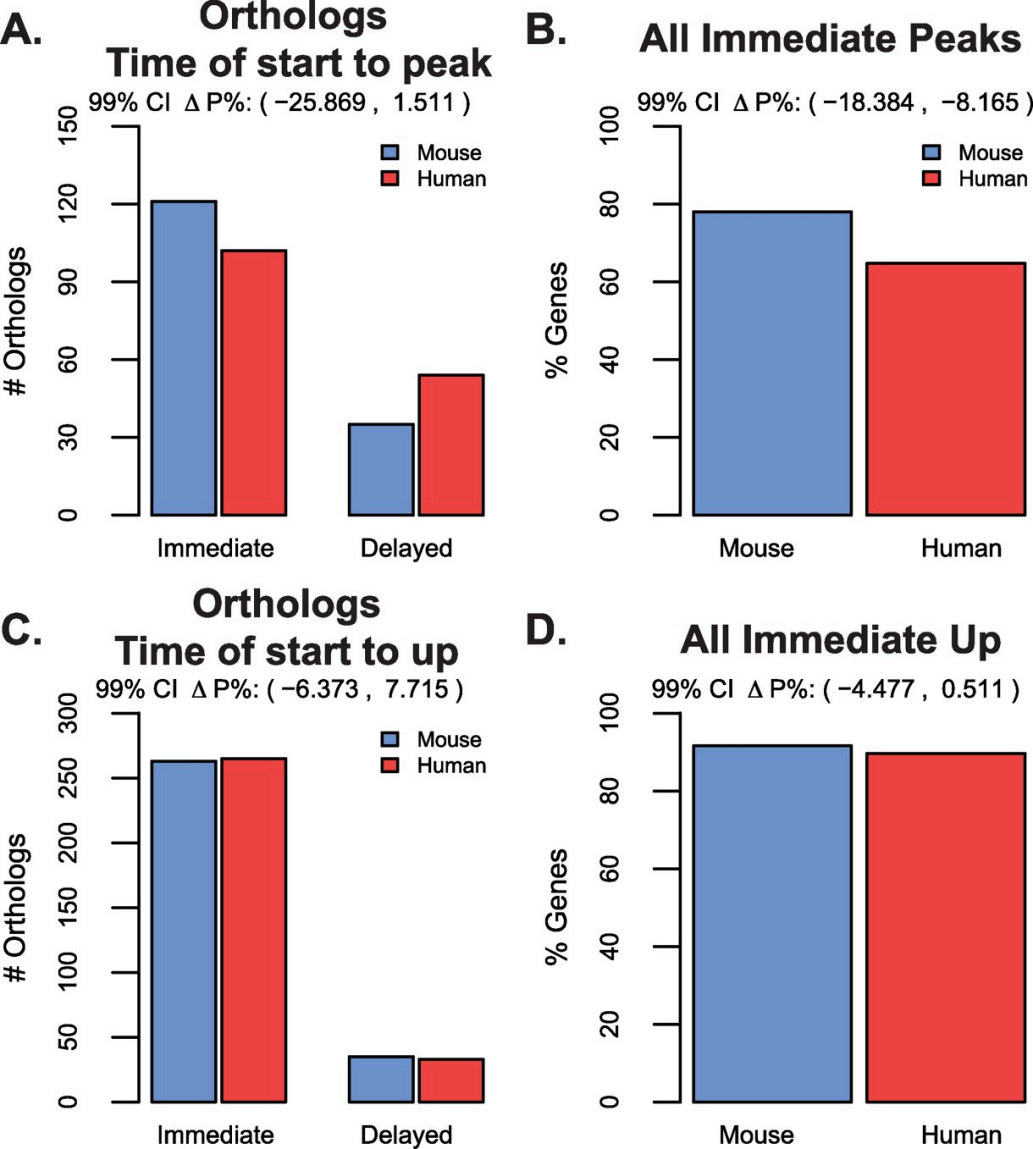
Immediate versus delayed dynamics are similar between mouse and human genes having a peak or upregulation. A) The number of ortholog peaks having the up trend starting immediately versus delayed in mouse and human. B) The percentage of all dynamic genes having an immediate peak in either mouse or human. C) Similar in structure to (A) for the number of orthologs having an having an immediate up trend. This broader categorization does include peaks. D) Similar in structure as (B) for all genes having upregulation in either mouse or human time courses.

As previously mentioned, there was no substantial difference between the percentage of genes that began their upward trends immediately versus later in the time course between mouse and human cells (91% versus 94%, respectively) (Fig 2B). We expanded this analysis to determine whether there were differences between species in the time the peaks began their upward trend, thus differentiating between an immediate dynamic peak versus a delayed peak. Interestingly, we discovered that there was no difference between species in the percent of genes having an immediate peak versus delayed peak for orthologs (Fig 5A; 99% CI on ΔP% -25.9% to 1.5%). Though when comparing over all peak genes, mouse cells did have approximately 13% fewer genes with a delayed peak (99% CI on ΔP% -18.4% to -8.2%; Fig 5B).

We then asked if this was true for all genes that were upregulated as a first response to differentiation (i.e. any gene that was immediately dynamic up or had delayed upregulation). We found no differences in the percentage of genes that go up immediately versus delayed across species, for either common dynamic orthologs or all genes that go up in either mouse and human cells (Fig 5C and Fig 5D). Again, we confirmed that these differences were not a result of sampling frequencies (Figure D-E in S1 Fig).

In the situation of a delayed peak in human versus mouse cells, the explanation for earlier peak times is clear. However, the vast majority of genes and orthologs showed no species-specific difference in the onset of upregulation, thus we hypothesized that the component responsible for the earlier peak times in mouse compared to human genes must be the rate at which genes are upregulated. The rate of change in gene expression was assessed by measuring the slopes of changes in gene expression over time in mouse and human cells.

**Fig 6:**
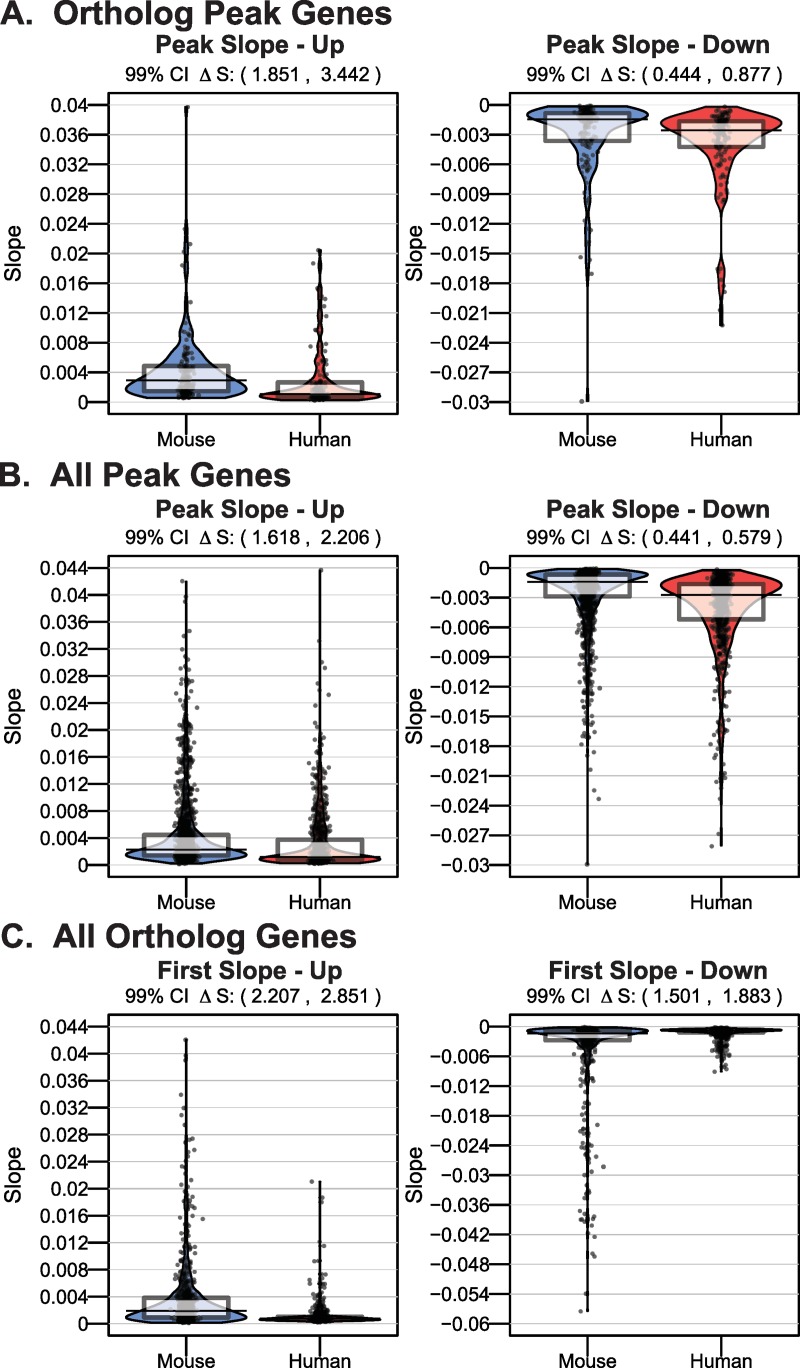
Rate of upregulation of gene expression is faster in mouse compared to human cells. A) For orthologs having a peak in both mouse and human cells, the slopes of the up trend (left) and down trends (right) of the peak are shown as smoothed densities (bean plots) with overlaid raw data, the median, and a box representing the interquartile range. B) Similar as (A) for all genes having a peak in either mouse or human cells. C) For all orthologs having an immediate up trend, the slope of the first breakpoint is shown in a smoothed density plot (left). For genes having an immediate down trend, the slope of the first breakpoint is shown (right).

To ensure that slopes were comparable across species and not biased by any species-specific differences in dynamic range of expression, we scaled each gene’s expression to be between 0 and 1 prior to running Trendy (Section 5 in S1 Text). Since the slope units are on the scaled expression, we compared slope magnitudes using the ratio of the median slope in mouse cells to the median slope in human cells (ΔS). A value larger than 1 indicates the slopes are larger in mouse cells, whereas values less than 1 indicates slopes are larger in human cells (Methods). When only assessing genes having peaks in both species, we measured notably steeper slopes in gene upregulation in mouse versus human cells, but not downward slope after peaks (Up slopes: ΔS = 2.697, 99% CI 1.851 to 3.442; Down slopes: ΔS = .5681, 99% CI 0.444 to 0.877; Fig 6A). We also found that when broadening the analysis to all peak genes in either species, the slopes leading up to mouse peaks were again steeper than those for human peaks (ΔS = 1.921, 99% CI 1.618 to 2.206; Fig 6B). The magnitude of down slopes after the peaks however were considerably smaller in mouse compared to human genes (ΔS = 0.519, 99% CI 0.441 to 0.579; Fig 6B).

We then expanded our analysis to consider all immediate dynamic changes in gene expression among orthologs in human and mouse cells to discern if the differences in rates of change in gene expression were specific to genes that exhibit peaked expression trends or also manifested in dynamic genes transcriptome-wide. Again, we found significantly faster rates of initial gene upregulation in mouse compared to human cells (ΔS = 2.499, 99% CI 2.207 to 2.851; Fig 6C), whereas the immediate down slopes were marginally steeper (ΔS = 1.674, 99% CI 1.501 to 1.883; Fig 6C). Again, our observations are preserved even in less frequently sampled mouse cells (Figure G–L in S1 Fig). Taken together, we found that changes in gene expression occurred more quickly in mouse compared to human pluripotent stem cells upon initial exposure to identical neural differentiation signals.

Recent studies have reported that intron versus exon counts can be used to assess transcriptional activity versus post-transcriptional regulation [29]. We mapped the intronic reads for our mouse and human time courses and used Trendy to obtain the expression pattern fits. Given our polyA procedure and sequencing depths we did not have sufficient coverage to perform a genome-wide analysis on the intronic reads for all genes, but we were able to identify some signals of interest. We fit Trendy to gene intron read counts and used the adjusted R^2^ > 0.2 threshold to identify 50 dynamic orthologs. To determine whether our intronic read counts on these 50 genes was sufficient, we first investigated the relationship between a singular change in intron reads versus the change in exon reads. We treated the first and last 10 time points as separate ‘conditions’ and calculated the difference in mean expression on the exon and intron reads for the orthologs. In both human and mouse time courses the change in exon counts was highly correlated to the change in intron counts indicting that changes in transcription were detectable (Figure A in S4 Fig). However, only 11 genes among those having sufficient coverage were also found to be common orthologs that had a peak in human and mouse cells, though the subset included genes involved in differentiation (S2 File). To address whether increased transcriptional activity is responsible for the faster slopes in mouse compared to human, we compared the intronic read slopes between mouse and human cells for genes that also were upregulated in either mouse or human exon reads. Mouse upregulated intron slopes were significantly steeper in mouse cells (Figure B in S4 Fig), which suggests faster accumulation of immature transcripts due to more rapid transcriptional responses rather than decreased degradation.

Lastly, since these experiments measured the first wave of early changes in gene expression during the first 10 hours of differentiation, we also applied our slope analysis to a longer time course to test if the difference in slope magnitudes we observed across species was conserved over longer developmental time frames. We analyzed neural differentiation in two additional datasets: a 3 week time course for mouse pluripotent stem cells and 6 week time course for human pluripotent stem cells [14]. When we applied our analysis to the longer developmental time frame datasets, we found 219 peak ortholog genes and confirmed that the rates of change in gene expression were significantly greater in mouse versus human cells during peak upregulation (ΔS = 1.667, 99% CI 1.315 to 2.234) but not significantly different in downregulation after peaks (ΔS = 1.359, 99% CI 0.996 to 2.021; S5 Fig). These results suggest that differences in gene upregulation kinetics may influence differentiation rates over longer periods of developmental time.

## Discussion

In this study, we compared changes in gene expression at the onset of differentiation between mouse and human pluripotent stem cells. Using an automated liquid-handling robotics system and subsequent RNA-seq analysis, we were able to track changes in gene expression at the uncharted, immediate-early stages of differentiation and at unprecedented temporal resolution. The segmented regression (breakpoint) model approach implemented here is both powerful and flexible by estimating gene expression changes and trends without a priori defining trend categories. The method implemented in the Trendy R package output the best fit breakpoint model for each gene, summarizing quantitative measures of breakpoint times and slopes of the connecting segments which we were able to compare across species in our statistical analysis. We uncovered (1) a previously unidentified highly dynamic gene expression landscape within the first twenty minutes of mouse and thirty minutes of human stem cell differentiation, (2) gene expression patterns were protracted in human compared to mouse cells, (3) the time of initial gene upregulation off of baseline was not different between human and mouse genes, and, most interestingly, (4) changes in gene expression occurred significantly faster in mouse compared to human pluripotent stem cells as measured by the slopes of gene upregulation. Taken together, these results identify species-specific differences in the kinetics of gene expression responses to differentiation cues from the earliest stages of development.

Time courses of pluripotent stem cell differentiation have typically involved daily sampling frequencies, and rarely as frequently as every 6 hours [30]. While these brief interspersed glimpses reflect snapshots of cell maturity, they are unable to capture the kinetics of gene expression changes at the onset of differentiation. The species-specific differences in transcriptome-level response rates to differentiation initiation we report in this study are consistent with a causative effect on the subsequent timing of lineage specification reported in other studies over longer time scales [4,14,31,32]. We did not observe correlation between mouse and human cells in the timing of expression changes, which corresponds to previous findings of variation in sequential orders of gene expression across species [33–38]. Although our study was restricted to human and mouse pluripotent stem cells, studies that examine developmental timing across other species are also consistent with a model in which changes in transcriptome dynamics could contribute to differences in developmental rates [7,8,11,12,39–41].

We also found enrichment of immediate-early response genes in our peak genes corresponding to species-specific differences in response to neural induction media. We consider the peak genes identified in our study that are not yet classified as IEG in the literature as IEG candidates and contend that the segmented regression framework here could easily allow identification of novel IEGs when applied to additional early time course experiments by identifying immediately dynamic genes and calculating the fold-change of the response as the amplitude of the expression trend. Given the short 10-hour time-frame focus in our study we focused mainly on peak trends, however this framework is also able to identify cyclic trends and could be expanded to allow for non-linear segmented modelling.

Our data in this study does not support a model of delayed signal delivery to the nucleus (model 1), but rather a difference in the velocity of transcriptional upregulation across species (model 2). First, we did not observe an overall increase in percentage of genes with a delayed onset of up- or downregulation changes in human compared to mouse genes (Figs 2, 3 & 5). Second, differences in the shape of gene peaks caused by differences in slope, rather than a delayed onset of identical peak shapes, were responsible for the divergence in peak times across species (Figs 4 & 6). Indeed, a signal reaching the nucleus more slowly would have manifested in the delayed onset of similar first up-slopes. In contrast, we report here differences in slope magnitudes within similar start times in mouse and human cells, consistent with divergent kinetics of transcription across species. Moreover, changes in gene expression occurred much more quickly in mouse compared to human cells in up-slope magnitudes, whereas both species shared more similar down-slope inclines. This observation is suggestive that it is the speed of transcription that is different across species rather than differences in mRNA degradation, which is also consistent with our intron/exon analysis where increases of intron accumulation was greater in mouse compared to human cells for certain genes of interest during differentiation. However, our polyA selection of transcripts led to low intron coverage in this experiment which was insufficient to make broader claims for the mechanisms behind faster accumulation of transcripts in mouse compared to human cells. Future experimentation will be necessary to directly measure and compare transcription burst rates versus mRNA degradation rates across species during development. However, taken together, our results are consistent with a model in which signals in the nucleus result in species-specific differences in transcriptional upregulation kinetics of response genes.

Several possible causes may explain the differences in transcriptional response kinetics observed in this study. Transcription factor binding affinities, co-factor recruitment stringencies, numbers of regulatory pathway signals, and/or fundamental RNA polymerase kinetics may vary in human and mouse stem cells, although there is little evidence to support the latter [42]. However, a remarkable amount of differences in transcription factor binding sites and gene regulation mechanisms have been reported between mouse and human stem cells [37,43–48], which may strongly influence rates of gene expression responses, even when given identical differentiation signals.

Differences in gene expression kinetics are unlikely to be solely responsible for the tremendous differences in developmental time across species. Mammals vary in size by an astounding eight orders of magnitude, and vary in gestation length by over 50-fold [49]. The mechanisms of the developmental clock must therefore coordinate across vast differences of time and scale, and may thus span multiple biological processes, one of which may involve the kinetics of signal-induced gene regulation.

Importantly, the retention of a slow developmental clock by human pluripotent stem cells presents a significant challenge for the development of pluripotent stem cell-derived therapies [50,51]. Current protocols to derive many cell types of potential importance for cell replacement therapies require extensive periods of time [4,52,53]. Producing the desired cell types with the necessary level of physiological maturity in clinically-relevant time frames will be essential if the therapy is aimed at being patient-specific [52–55]. Thus, understanding the developmental clock and identifying ways to accelerate it would have a broad impact on improving the therapeutic value of human pluripotent stem cells.

In summary, we measured transcriptome-wide expression at the scale of minutes in the immediate-early response to neural differentiation factors in human and mouse pluripotent stem cells. Understanding what controls species-specific rates of differentiations is a step forward in defining the developmental clock, which is an essential piece to overcoming obstacles in developing pluripotent stem cell therapies. Our findings suggest that the differences in developmental rates observed across species are in part due to differences in transcriptional response rates to differentiation factors.

## Materials & methods

### Ethics statement

All experiments described in this study were approved by the ethics committee with IRB Approval Number: SC-2015-0010. The H1 hES cells are registered in the NIH Human Embryonic Stem Cell Registry with the Approval Number NIHhESC-10-0043.

### Cell culture

EGFP-positive H1 and mEpiS cells were maintained as previously described [14]. H1 cells were cultured on matrigel-coated plates in E8 medium (Thermo Fisher Scientific, USA) and mEpiS cells were cultured on MEF feeder cells in TSS (DMEM/F12 (Thermo Fisher Scientific, USA), using 20% knockout serum replacement (Thermo Fisher Scientific, USA), 0.18 mM B-mercaptoethanol (Sigma, USA), 1 X non-essential amino acids (Thermo Fisher Scientific, USA), 2 mM L-glutamine (Sigma, USA), 7.5 ng/mL activin A (R&D Systems, USA), and 5 ng/mL bFGF (R&D Systems, USA)).

Cell suspensions were seeded in pluripotent media prior to neural induction. H1 cells were lifted in 0.5uM EDTA and cells were transferred to the robotic system at 4°C under sterile conditions in E8 medium. mEpi3S cells were dissociated using TryPLE, however were first depleted of MEFs by MACS separation using feeder cell removal beads according to the manufacturer’s instructions (Miltenyl Biotec, Germany). Cells were then resuspended in TSS medium and transferred to the robotic system under sterile conditions at 4°C. Mouse and human cells were kept at 4°C until either 2.5X10^5^ or 5.28X10^5^ cells were seeded into 12- or 6-well matrigel-coated plates, respectively, and were shuttled to 37°C incubators and attached under pluripotent conditions (H1 cells in E8, mEpiS cells in MEF-conditioned TSS with 20ng/mL mFGF) until neural induction.

### Automated cell culture and liquid handling system

A Freedom Evo200 liquid handling robot was located in a BigNeat custom controlled environmental enclosure. The time course was automated using a custom-built automated platform (Tecan, BigNeat and LiConiC). The platform was programmed using TECAN Evoware Plus software, and various custom software drivers to control peripheral devices were used. The enclosure maintained an environment of 30°C, with 5% CO2 and 80–90% relative humidity (RH) during all liquid handling steps.

Integrated peripheral devices were utilized to perform automated tissue culture and generate cellular lysates. These devices included a Liconic STX500 incubator (maintained at 37°C, 5% CO2 and 80–90% RH), a Liconic STX200-DF freezer (for sample storage after cell lysis), a Tecan Te-Shake shaker, and a Tecan Tilt Carrier for media removal.

### Neural induction and sampling for RNA-seq

At time 0, pluripotent media were removed from the wells of the previously seeded 12- or 6-well matrigel plates, cells were washed with 1 X PBS (Thermo Fisher Scientific, USA), 37°C pre-warmed neural induction medium was added (DF3S (Thermo Fisher Scientific, USA) supplemented with 1 X N2 supplement (Thermo Fisher Scientific, USA), 1 X B27 supplement (Thermo Fisher Scientific, USA), and 100 ng/μL of mNoggin (R&D Systems, USA)), as previously described [14], and cells were shuttled back to the 37°C incubator. The switch to neural induction media for each multiwell plate was staggered and timed to establish the proper feeding and collection times for each well. At the indicated time-points, the appropriate multiwell plates were shuttled out of the incubator and cells were lysed at either 4- or 10-minute intervals (+/- 2 seconds as calibrated according to the time of neural induction medium addition) for mouse and human cells, respectively. Neural induction medium was removed, cells were washed with 1 X PBS, lysed in 700μL RLT-Plus Buffer (Qiagen, Germany), and plates were shuttled to -20°C until all samples could be processed for RNA-seq together. In the control experiment described for S2 Fig, hES cells were exposed to Essential 8 (Thermo Fisher Scientific, USA) pluripotent maintenance medium rather than differentiation medium.

### Sample processing and RNA-seq pipeline

RNA-seq samples were processed from cell lysates as previously described [56]. Briefly, total RNA was purified from RLT-Plus Buffer using RNeasy Plus Mini Kits (Qiagen, Germany). The Ligation-Mediated Sequencing (LM-Seq) protocol was used to prepare and index all cDNA libraries [56]. Seven to 48 uniquely indexed samples were pooled per lane on an Illumina HiSeq 2500 or 3000 with a single 51 base pair read and a 10 base pair index read. Post-sequence reads were initially processed by trimming adapter sequences and aligned to mm10 or hg19 libraries using bowtie (v0.12.9) [57]. Sample qualities were screened for appropriate read depth and transcriptome alignment ratios; only samples with at least 50% alignment rates were retained for the analysis removing six time-points from the mouse time course (originally 151 time-points) and zero from the human experiment (originally 61 time-points). Excluded samples were not correlated to plate or well positional bias on the robotic system. For each species, expected counts (EC) expression measures were generated by RSEM (v1.2.3) [58]. Due to their variable abundance, mitochondrial genes were removed prior to normalization and downstream analysis. Expected counts of human samples sequenced across lanes for each time-point were added together for further analysis.

### Intron read mapping

To map intron reads, we adapted our previous alignment pipelines as follows. We first built an expanded transcriptome reference using hg38 (rather than hg19), and incorporated both NM (mRNA) and NR (non-coding RNA) transcripts. Then we built an ‘intronic’ transcriptome reference, with a single entry for each gene in the “mature” transcriptome reference. Each entry contained the full genomic sequence from the most 5’ TSS through the most 3’ UTR of all annotated transcripts for that gene. We applied our usual filters to trim low quality base calls and adapter sequences. We ran RSEM-1.3.0, using bowtie2 (v2.3.5.1) against the new mature-transcript reference [59]. From there we collected all reads with no reported alignment to the mature-transcript reference or whose primary alignment (per RSEM’s call to bowtie2) had an edit distance of three or more from the transcript reference, as indicated by the “NM:i” field in the SAM output. We mapped these remaining reads to the intronic reference using bowtie2 (v2.3.5.1). For this second run of bowtie2, in the case of multimapping alignments, the count was assigned to the primary alignment designated by bowtie2.

### Trendy analysis

After quality control the mouse and human RNA-seq datasets had 145 and 61 samples, respectively. Each dataset was normalized using median ratio normalization proposed by Anders and Huber 2010 [60] using the MedianNorm function in the EBSeq R package [61]. In each dataset, genes with an average normalized expression across all samples smaller than 10 were removed prior to further analysis. For the remaining genes, each of their expression profiles across samples was scaled between 0 and 1 as:

Xi,j=(Yi,j−minjYi,j)(maxjYi,j−minjYi,j)

where *Y_i,j_* represents the normalized expression of gene *i* in sample *j*.

The segmented models were fit with expression *X_i,j_* as a function of time *t*. For the mouse experiment *t* = (0,4,8,…,600) (the six missing times failing quality control were minutes 60, 132, 452, 492, 464, and 512) and for the human dataset *t* = (0,10,20,…,600). For notation, let *t* = (*t*_1_,*t*_2_,…,*t_N_*) for either experiment with N = 145 in mouse and N = 61 in human. The segmented regression model for gene *i* having a single breakpoint is:

$$X_{i,j}=\beta_{i,0}+\beta_{i,1}t_{j}I(t_{j}:t_{j}\geq t_{1},t_{j}\leq b_{i,1})+\beta_{i,2}{(t}_{j}-b_{i,1})I(t_{j}:t_{j}>b_{i,1},t_{j}\leq t_{N})$$

Where *b*_*i*,1_ is the breakpoint and *β*_*i*,1_ is the slope of the first segment prior to the breakpoints and *β*_*i*,2_ is the slope of the second segments after the breakpoint. The number of segments is always one plus the number of breakpoints. For each gene we fit a model having no breakpoints (standard linear regression) and then extended the model above to accommodate fitting five additional models each having the number of breakpoints from 1 to 5. For a breakpoint model fit to be valid, each segment was required to contain a minimum of five or three consecutive time-points representing 20 minutes in the mouse time course and 30 minutes in the human, respectively (Section 3 in S1 Text). Thus, Trendy assigned an initial up or down trend to genes that had an up or down response occurring within the first 20 minutes in mouse cells or 30 minutes in human cells. In this paper, we classified these genes as being ‘immediately’ dynamic. Genes that had an up or down response occurring after the first 20 or 30 minutes for mouse or human cells were classified as having a “delayed” dynamic.

The best breakpoint model was selected as the one having the lowest Bayesian information criterion (BIC). While every gene has a model fit, here we focused only on those considered having a ‘good’ fit, which we defined as genes having a fit with an adjusted R^2^ > 0.2. Only these genes were further considered in the analysis and for simplicity were referred to ‘dynamic’ genes. The cutoff was determined based on a permutation procedure (Section 2 in S1 Text). Each segment of the breakpoint regression model was assigned as “up”, “down” or “no-change”; the individual segment p-value threshold parameter for this characterization was set to 0.2. The human control experiment was fit using the same parameters and criteria listed above for the human neural differentiation experiment. Trendy was run on the intron reads using the same parameters as described above. The reduced mouse time course using every 3rd mouse sample (in S1 Fig), was run with the same fit parameters as the original 4 minute mouse time course. The human and mouse week-long time course experiments were fit with similar models as described, however since there were fewer samples (16 for human and 36 for mouse) and sampled at equal frequencies, we required segments to have a minimum of three consecutive time-points for both time courses. The criteria to have a dynamic fit was raised to R^2^ > 0.5 based on our permutation procedure (Section 2 in S1 Text). Exploration of all fitted expression patterns using Trendy’s interactive shiny application is available at https://github.com/rhondabacher/RobotNeuralDiffPaper.

### Pattern classification

Peak genes were classified as those having either a consecutive “up” and “down” segment along the time course experiment, including cyclic genes, or those having an “up”, “no-change”, “down” pattern. Genes displaying distinct patterns shown in Fig 1B and provided in S1 Table were defined as follows: delayed peak: same-up-down or same-up-same-down, delayed dip: same-down-up or same-down-same-up, delayed up: same-up, delayed down: same-down, immediate on: up-same, immediate-off: down-same, dip: down-up or down-same-up, peak: up-down or up-same-down, monotonic up: up, monotonic down: down, cyclic: any genes not in the previous categories having at least two disconnected up or down segments, for example, up-down-up, up-down-down-up, and up-same-down-up were all considered cyclic for classification in S1 Table.

### Statistical analysis

All analyses were performed in R version 3.6.0 [62]. The Trendy R package (v1.7.3) was used to fit the segmented regression models to each gene, select the optimal model and then reported the breakpoint times and segment slopes in each dataset [22]. Orthologous mouse and human genes were downloaded using the biomaRt R package (v2.40.0) [63]. The distribution of breakpoint times, peak times, and segment slopes were plot using both histograms and smoothed densities (bean plots) with overlaid raw data, the mean, and a box of the interquartile range. The combined bean plots were created using the pirateplot function in the yarrr R package (v0.1.5) [64]. When comparing the ortholog peaks, only the first peak was used for peak time and slope calculations. Genes having multiple peaks (cyclic trend) were not the focus of this study. For comparing the distribution of peak times, the wilcox.test() function in R was used to obtain 99% confidence intervals on the median shift in the human peak time distribution compared to the mouse peak time distribution (ΔM). For orthologs, the paired Wilcoxon test was used with option paired = TRUE. For comparing the percent of genes in human cells versus the percent of genes in mouse cells having an immediate versus delayed peak or upregulation, 99% confidence intervals were calculated from a two-proportions z-test using the prop.test() function in R and multiplying the resulting intervals by 100%. For comparing the slope magnitudes, we used the np.re() function in the pairwiseCI R package (v.1-27) [65] with method = Median.ratio to obtain 99% confidence intervals on the ratio of the median slope in mouse cells to the median slope in human cells. This provides a nonparametric estimate of the relative difference in the slopes (ΔS).

Analyses to determine functional enrichment of orthologs having a peak and genes having dynamic expression in both the human differentiation and control experiment were performed using the GSEA algorithm on the GO biological processes collection in MSigDB (v7.0 MSigDB, FDR q-value < .001) [66,67]. For transcription factor enrichment we used the web-based tool Enrichr on the TRRUST Transcription Factors 2019 annotations [68,69]. Figure A in S1 Fig was generated with the R package ggsunburst v0.0.10 [70].

## Supporting information

S1 Text 1 –Evaluating the appropriateness of piece-wise linear model fits
2 –Choice of threshold for dynamic Trendy fit.
3 –Choice of minimum segment length parameter.
4 –Detectable resolution of dynamic expression by Trendy.
5 –Scaling data to obtain comparable slope magnitudes.
(DOCX)

S1 Table Sheets 1 and 2 contain the breakdown of the number of genes having the patterns described in Fig 1B for both mouse and human time course experiments. Sheets 3 contains the lists of genes having each pattern in mouse cells sampled every four minutes. Sheet 4 contains the lists of genes having each pattern in human cells sampled every 10 minutes.
(XLSX)

S2 Table Summaries of the Trendy fitted model for each gene having a dynamic trend in both human and mouse cells. The information includes: segment slopes, segment direction, segment p-value, time of breakpoint, and trend direction for each time-point.
(XLSX)

S3 Table Summaries of the Trendy fitted model for each gene having a dynamic trend in either species. (XLSX)

S4 Table Enrichment of transcription factors for genes having a dynamic trend in both mouse and human cells. (TXT)

S5 Table Biological processes enriched in sets of genes from the Human control and Human differentiation experiments as shown and defined in S2 Fig. (XLSX)

S6 Table Summaries of the Trendy fitted model for each ortholog having a peak in both mouse and human cells. (XLSX)

S7 Table Biological processes enriched in the set of orthologs having a peak in both species. (XLSX)

S8 Table Summaries of the Trendy fitted model for each ortholog having a peak in either mouse and human cells. (XLSX)

S1 File Scatter plots of the immediate early genes (IEG) having a peak in both the human and mouse time courses. (PDF)

S2 File List of orthologs having a peak on intron reads in both mouse and human cells. (TXT)

S3 File Raw read counts for all experiments analyzed. (XLSX)

S1 Fig Different sampling frequencies between human and mouse cells did not bias the measured times of initial changes in gene expression, earlier breakpoints, or steeper slopes in mouse compared to human cells. We re-ran Trendy and our entire analysis pipeline using every 3^rd^ mouse sample in the time course to effectively simulate a reduced sampling rate to levels comparable to the human time course (lengthened from 4 minutes to every 12 minutes in mouse compared to 10 minutes in human cells). Using this modified sampling frequency, we compared the time of initial onset of Up/Down trends in gene expression (A), numbers of monotonic genes (B), and time to first breakpoint (C) of common ortholog genes between mouse and human cells. The same analysis was carried out on all dynamic human and mouse genes even if the same gene was not fitted with a dynamic trend in the other species (D, E, and F, respectively). Similarly, gene expression peak times (G) and slopes Up (H) and Down (I) from the peak were measured in ortholog genes having a peak in both mouse and human cells, as well as for genes with a peak in at least one species (J, K, & L, respectively).
(TIF)

S2 Fig Dynamic genes identified by Trendy are involved in development. A) Among all 4332 human genes identified by Trendy, 1634 are also dynamic (adjusted R^2^ > 0.2) in the feeding control RNA-seq time course. Similarity of trends was based on genes initial response direction; genes starting in the same direction were considered similar and genes with trends in the opposite direction were considered opposite. B) The breakpoint distribution of the differentiation and control experiments with times where multiple genes had changes in expression highlighted. C.) Top GO terms from a gene-overlap enrichment analysis for the genes indicated in the sets from B are shown. None of the synchronized breaktimes in the control were enriched for differentiation processes and instead had strong cell cycle signals. Given that those cells are not responding to differentiation media, cell cycle is the dominant biological process we detect.
(TIF)

S3 Fig Variation in the sequential order of mouse and human peak times. For all orthologs having at least one peak in both mouse and human cells, the time of the first (or only) peak is shown with the Mouse peak time on the x-axis and the Human peak time on the y-axis.
(TIF)

S4 Fig Intron reads give insight intro transcriptional activity. A) Treating the first and last ten time-points as a ‘condition’, the mean change in exon reads versus mean change in intron reads is shown for the human and mouse time courses. The high correlation indicates transcriptional activity is detectable. B) The intronic read slopes between mouse and human cells for genes that also were upregulated in either mouse or human exonic reads.
(TIF)

S5 Fig Species-specific differences in rates of change in gene expression are conserved in longer neural differentiation time courses. A) For all ortholog peak genes, the slopes of the up-trend (top) and down trend (bottom) are shown in a boxplot for the 10 hour robot time course. B) The same plots are shown for ortholog peaks in mouse and human time courses of three and six weeks, respectively, in Barry et al., 2017.
(TIF)
