# Supplementary material for: Automated minute scale RNA-seq of pluripotent stem cell differentiation reveals early divergence of human and mouse gene expression kinetics
Source: PLoS Comput Biol. 2019 Dec 9;15(12):e1007543. doi: 10.1371/journal.pcbi.1007543 (PMC6922475; doi:10.1371/journal.pcbi.1007543)
Supplement: S1 Text — 1 –Evaluating the appropriateness of piece-wise linear model fits 2 –Choice of threshold for dynamic Trendy fit. 3 –Choice of minimum segment length parameter. 4 –Detectable resolution of dynamic expression by Trendy. 5 –Scaling data to obtain comparable slope magnitudes. (DOCX) [file pcbi.1007543.s001.docx]

**S1 Text**

**Section 1. Evaluating the appropriateness of piece-wise linear model fits**

We evaluated the appropriateness of the break-point regression modelling approach used in Trendy at capturing overall trends by comparing to polynomial regression. For the mouse and human time-courses, after excluding genes with mean expression smaller than 10, we fit a polynomial regression model of degree five and assessed the goodness of fit between the polynomial and Trendy models for each gene based on its adjusted R^2^.

The adjusted R^2^ distributions for the polynomial model fit conditional on the Trendy fit for the mouse time-course are shown below. Of 7,421 genes with an adjusted R^2^ smaller than 0.2 in the Trendy fit (top left), 454 had an R^2^ > 0.2 for the polynomial fit (top right, highlighted in blue). However, of the 3,721 genes that have an adjusted R^2^ larger than the 0.2 cutoff in the Trendy fit (bottom left), there are 353 genes below 0.2 in the polynomial fit (bottom right, highlighted in blue).

Similarly, for the human time-course, we found 393 genes having an R^2^ > 0.2 in the polynomial fit (top right, highlighted in blue) out of the 8,425 genes that originally had an adjusted R^2^ smaller than 0.2 in the Trendy fit (top left). However, among the 4332 genes with an R^2^ above 0.2 in Trendy (bottom left), 442 of those genes had R^2^ < 0.2 in the polynomial fit (bottom right, , highlighted in blue).

Overall, we do not find evidence to support that polynomial modelling offers a significant advantage to capturing dynamic gene expression. In fact, the advantage of fitting a piece-wise linear model using Trendy is its flexibility to fit complex expression patterns, approximately as well as polynomial regression, while providing quantitative and practical summaries of expression patterns.

**Section 2. Choice of threshold for dynamic Trendy fit.**

Although Trendy provides a fit for all genes passing a mean expression threshold, not all fits are dynamic or good--as with any model. To identify genes that have dynamic gene expression across the time-course and to exclude genes that are constant or have noisy expression, we only consider genes having a Trendy fit with an adjusted R^2^ (goodness of fit) larger than a certain cut-off. Every experiment will have different levels of noise in the expression depending on technical factors, for example, sequencing depth and library quality. Thus, the optimal choice is experiment dependent.

To determine reasonable cutoffs that provided confidence in a gene’s dynamic trend without being overly restrictive we performed permutation procedures on each time-course experiment. For a given time-course, we shuffled the order of the samples 100 times and for each shuffle, we ran Trendy using the exact same parameter settings as on the original datasets for 100 randomly sampled genes. We evaluated the adjusted R^2^ distribution of the Trendy fits on the permuted data which represented the null hypothesis of no dynamics. Shown below are the distributions for the mouse and human differentiation experiments (note that we cut the axis at 1000). We chose an adjusted R^2^ of 0.2, as less than 1% of the genes in the human permutations achieved this threshold and we used the same parameter for both human and mouse.

Shown below are the distributions for the mouse and human week long time-courses in S4 Figure. We chose an adjusted R^2^ of 0.5 as less than 1% of permuted genes reached this value.

**Section 3. Choice of minimum segment length parameter.**

One of the main parameters for Trendy is the minimum number of segments required for a segment to be valid and used to prevent overfitting. Since the mouse and human time-courses were sampled at different frequencies 4min and 10min, respectively, then using the default value of segment length equal to 5 corresponds to requiring any initial segment to last at least 20 minutes in mouse and 50 minutes in human. As this may bias our ability to detect early changes in the human cells, we lowered the parameter of minimum segment length to 3 for the human time-course. A segment length of 3 corresponds to requiring a trend to last 30 minutes in human.

Although this is still not equal, we did re-run Trendy on a subset of the mouse time-course (every 3rd sample) such that sampling was effectively reduced to only every 12 minutes. The minimum segment length remained equal to 5 when running Trendy, and thus required a segment to be increasing for at least 60 minutes. As shown in S6 Figure, this does not affect our results regarding mouse cells having steeper segment slopes or earlier breakpoint times. The parameter setting and less frequent sampling does result in more genes appearing to show monotonic expression, however this is still fewer than observed in the human data requiring segments to only last 30 minutes (Figures B and E in S1 Figure).

**Section 4. Detectable resolution of dynamic expression by Trendy**

We define a gene as being ‘immediately’ dynamic if its first segment identified by Trendy is significantly increasing or decreasing. Given the sampling rates of 4 and 10 minutes, along with the minimum segment length parameter set to 5 and 3 for mouse and human cells, respectively, the definition of ‘immediately’ dynamic corresponds to a gene’s expression increasing or decreasing at any point *within* the first 20 minutes for mouse cells or 30 minutes for human cells. Thus, any change in expression occurring within this initial time-frame is considered immediate. If no change in expression occurs for at least 20 or 30 minutes, the gene is considered as having ‘delayed’ dynamics.

Here, Trendy uses the nearby finely sampled time points to capture the expression dynamics of each gene. However, there are limitations. For example, if a gene has steady expression for 8 minutes in mouse cells and then begins increasing until 20 minutes, we still consider that an immediately dynamic gene. For a finer resolution examination between consecutive time-points Trendy (and any statistical method) would require additional biological replicates at all specific timepoints.

**Section 5. Scaling data to obtain comparable slope magnitudes.**

Differences in dynamic range for gene expression is unknown for mouse and human cells. Consider the scenario in which a gene’s maximal expression value is 100 in mouse and the maximal value in human is 1000. Even if the gene is dynamic for the same period of time, the slope in mouse would appear smaller due to the differences in dynamic range. For this reason, we scaled the expression across the time-course for each gene to be between zero and one. We ran Trendy on the raw normalized data and found that, as expected, the slope magnitudes are different; however, all other results are preserved. The same number of genes pass the adjusted R^2^ threshold, and the trends are all exactly identical in both mouse and human time-courses. The trendy fits on the raw and scaled expression data are available at <https://github.com/rhondabacher/RobotSeq>.
