# Supplementary figures and images for: Automated minute scale RNA-seq of pluripotent stem cell differentiation reveals early divergence of human and mouse gene expression kinetics
Source: PLoS Comput Biol. 2019 Dec 9;15(12):e1007543. doi: 10.1371/journal.pcbi.1007543 (PMC6922475; doi:10.1371/journal.pcbi.1007543)

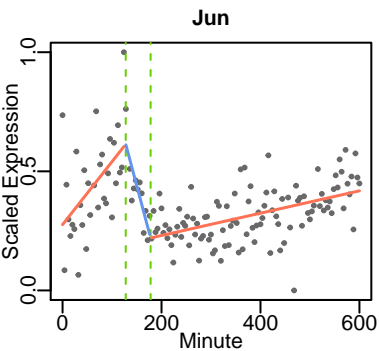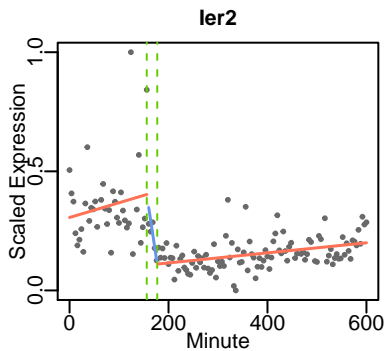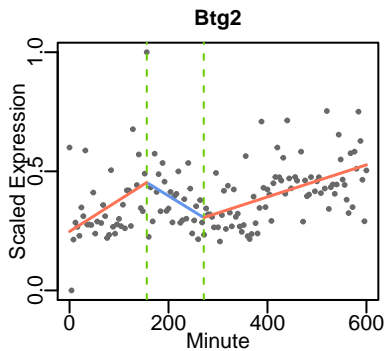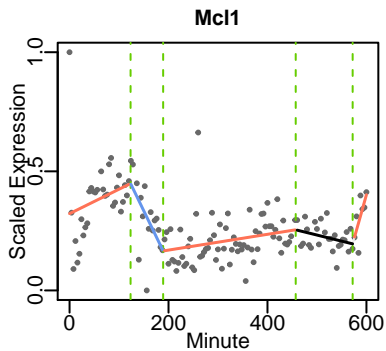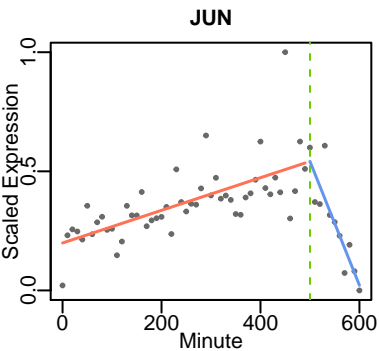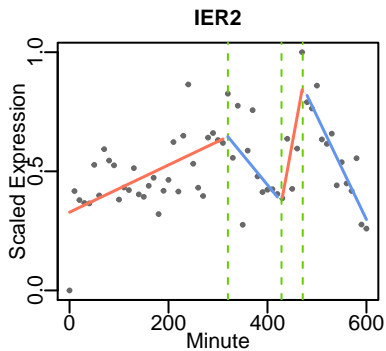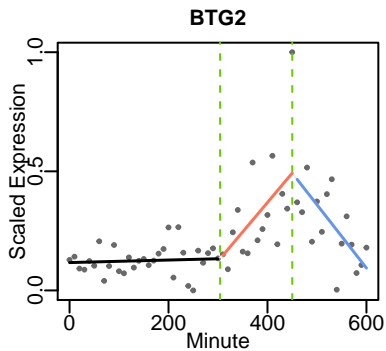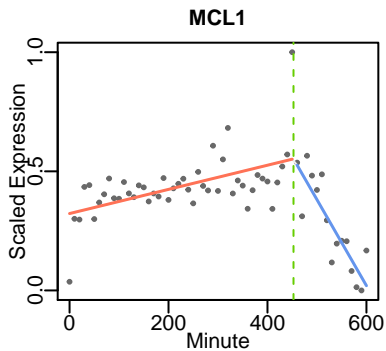

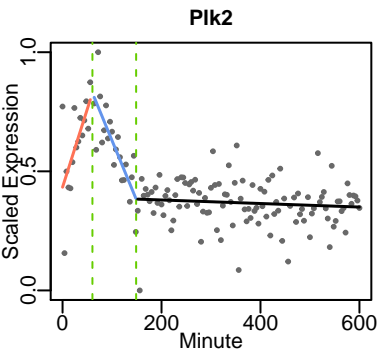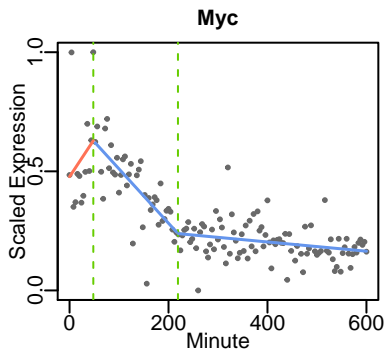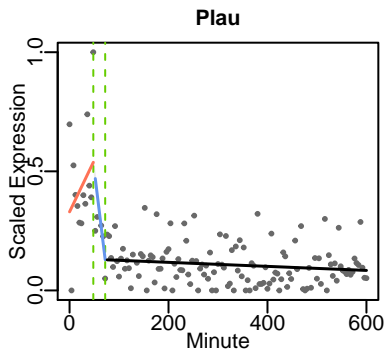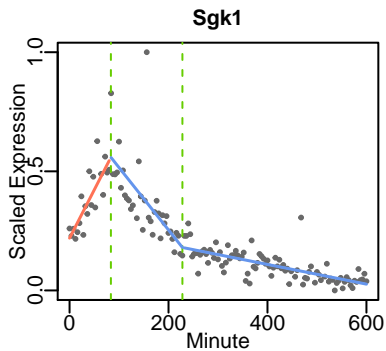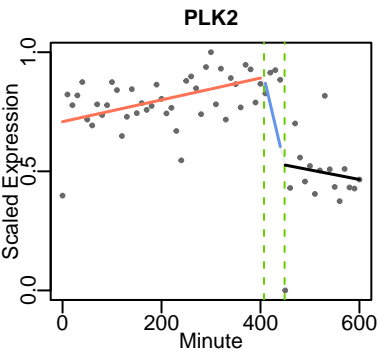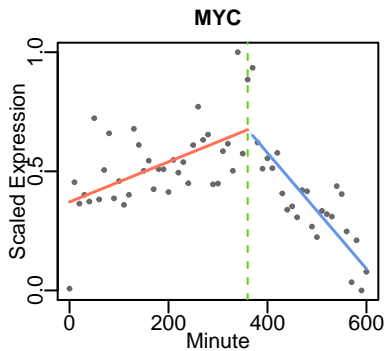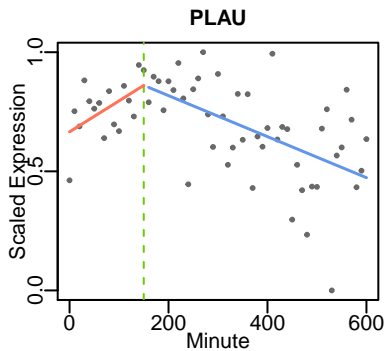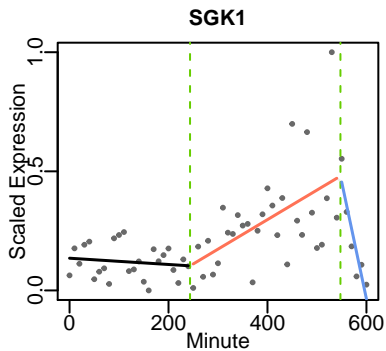

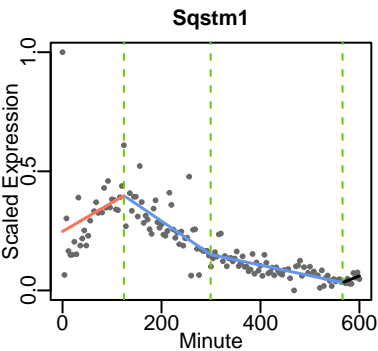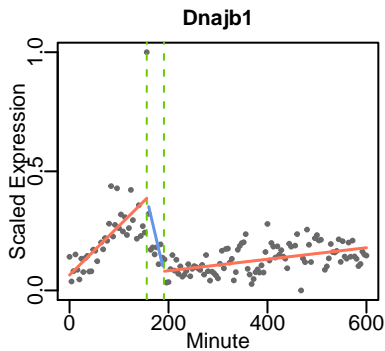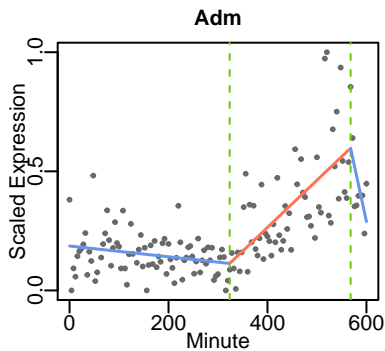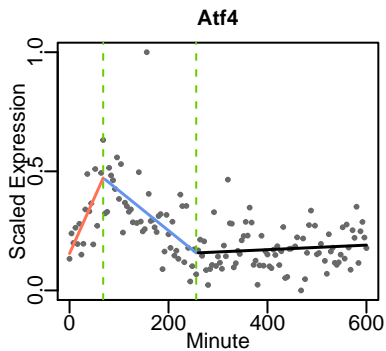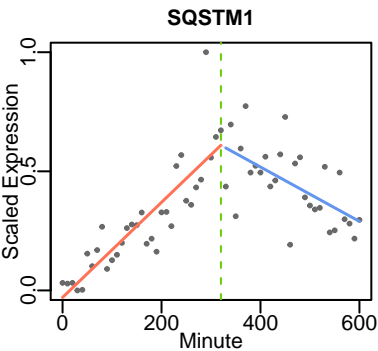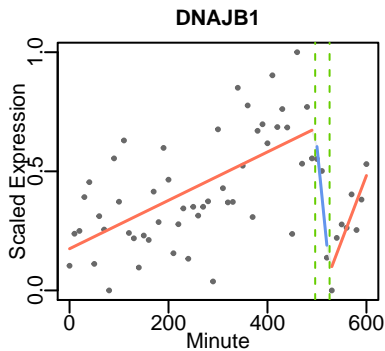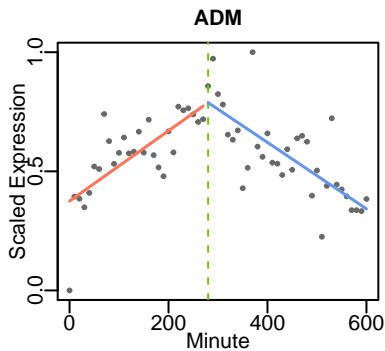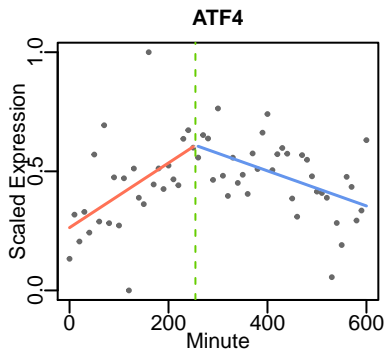

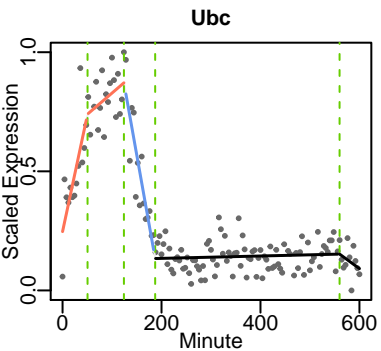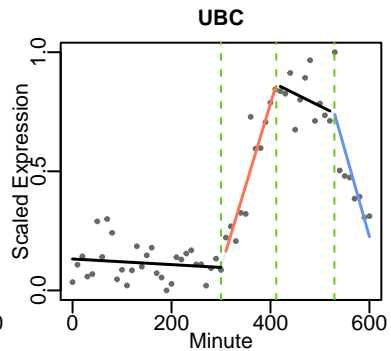

Supplement: S1 File — (PDF) [file pcbi.1007543.s010.pdf]

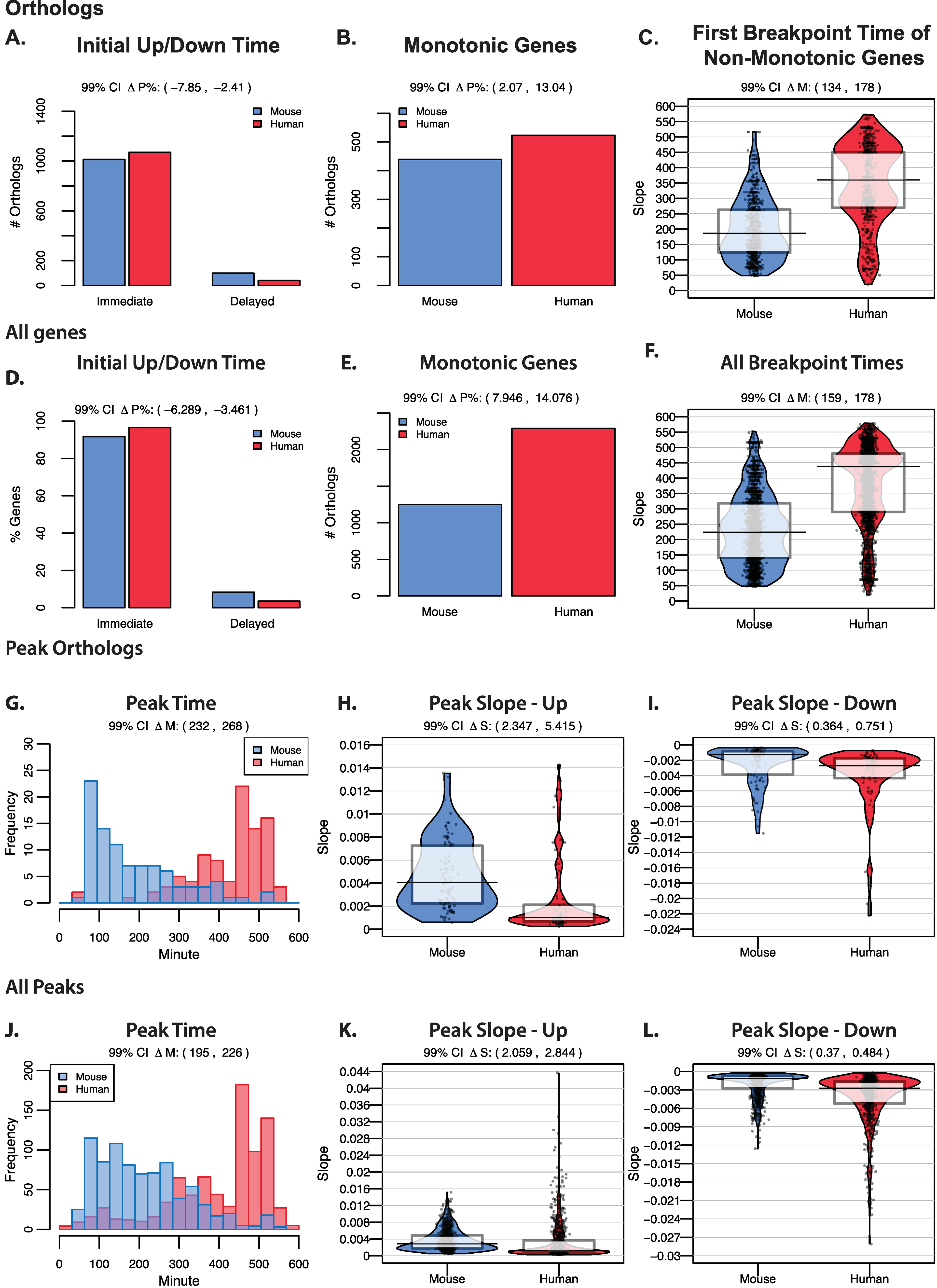

Supplement: S1 Fig — We re-ran Trendy and our entire analysis pipeline using every 3rd mouse sample in the time course to effectively simulate a reduced sampling rate to levels comparable to the human time course (lengthened from 4 minutes to every 12 minutes in mouse compared to 10 minutes in human cells). Using this modified sampling frequency, we compared the time of initial onset of Up/Down trends in gene expression (A), numbers of monotonic genes (B), and time to first breakpoint (C) of common ortholog genes between mouse and human cells. The same analysis was carried out on all dynamic human and mouse genes even if the same gene was not fitted with a dynamic trend in the other species (D, E, and F, respectively). Similarly, gene expression peak times (G) and slopes Up (H) and Down (I) from the peak were measured in ortholog genes having a peak in both mouse and human cells, as well as for genes with a peak in at least one species (J, K, & L, respectively). (TIF) [file pcbi.1007543.s013.tif]

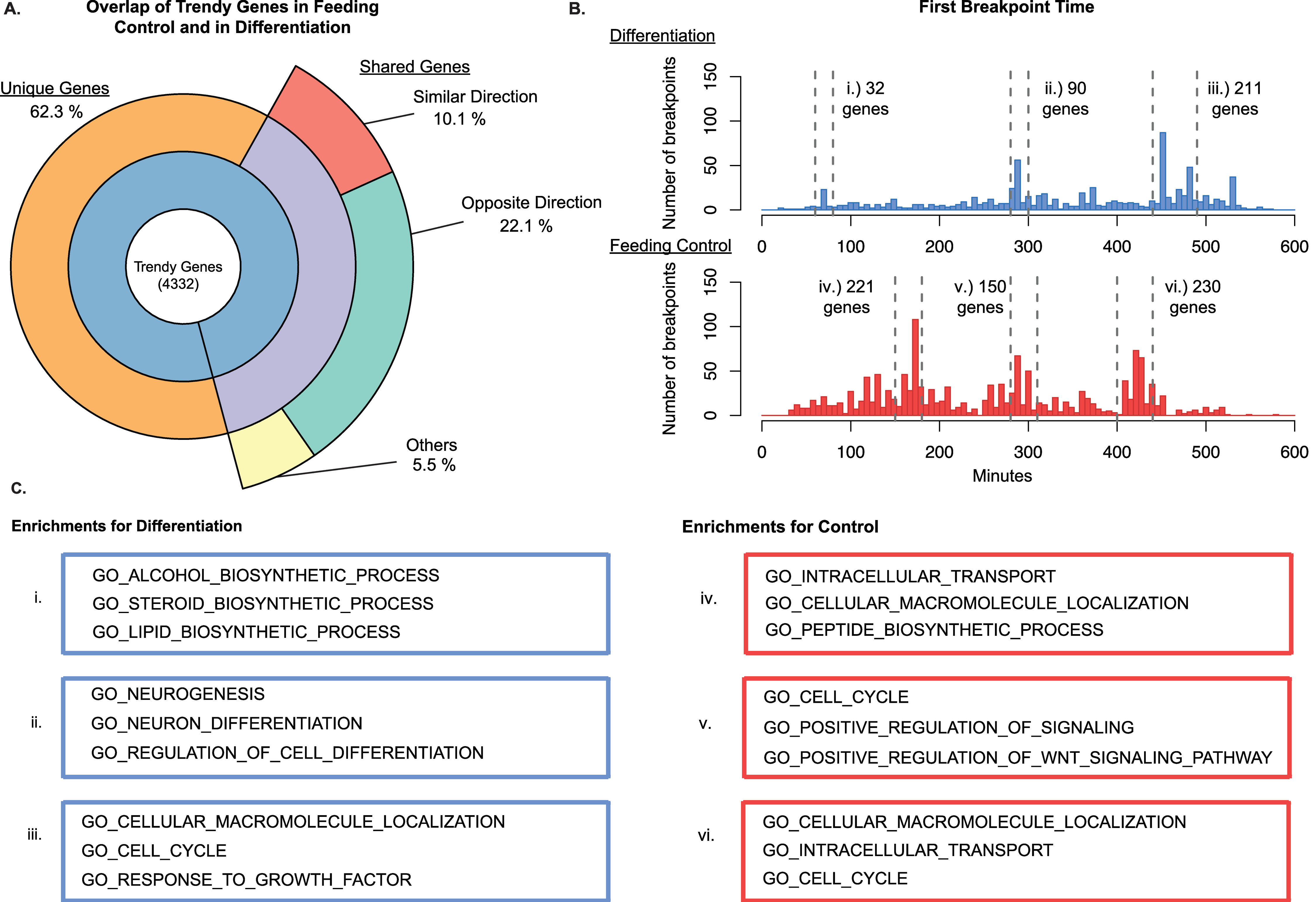

Supplement: S2 Fig — A) Among all 4332 human genes identified by Trendy, 1634 are also dynamic (adjusted R2 > 0.2) in the feeding control RNA-seq time course. Similarity of trends was based on genes initial response direction; genes starting in the same direction were considered similar and genes with trends in the opposite direction were considered opposite. B) The breakpoint distribution of the differentiation and control experiments with times where multiple genes had changes in expression highlighted. C.) Top GO terms from a gene-overlap enrichment analysis for the genes indicated in the sets from B are shown. None of the synchronized breaktimes in the control were enriched for differentiation processes and instead had strong cell cycle signals. Given that those cells are not responding to differentiation media, cell cycle is the dominant biological process we detect. (TIF) [file pcbi.1007543.s014.tif]

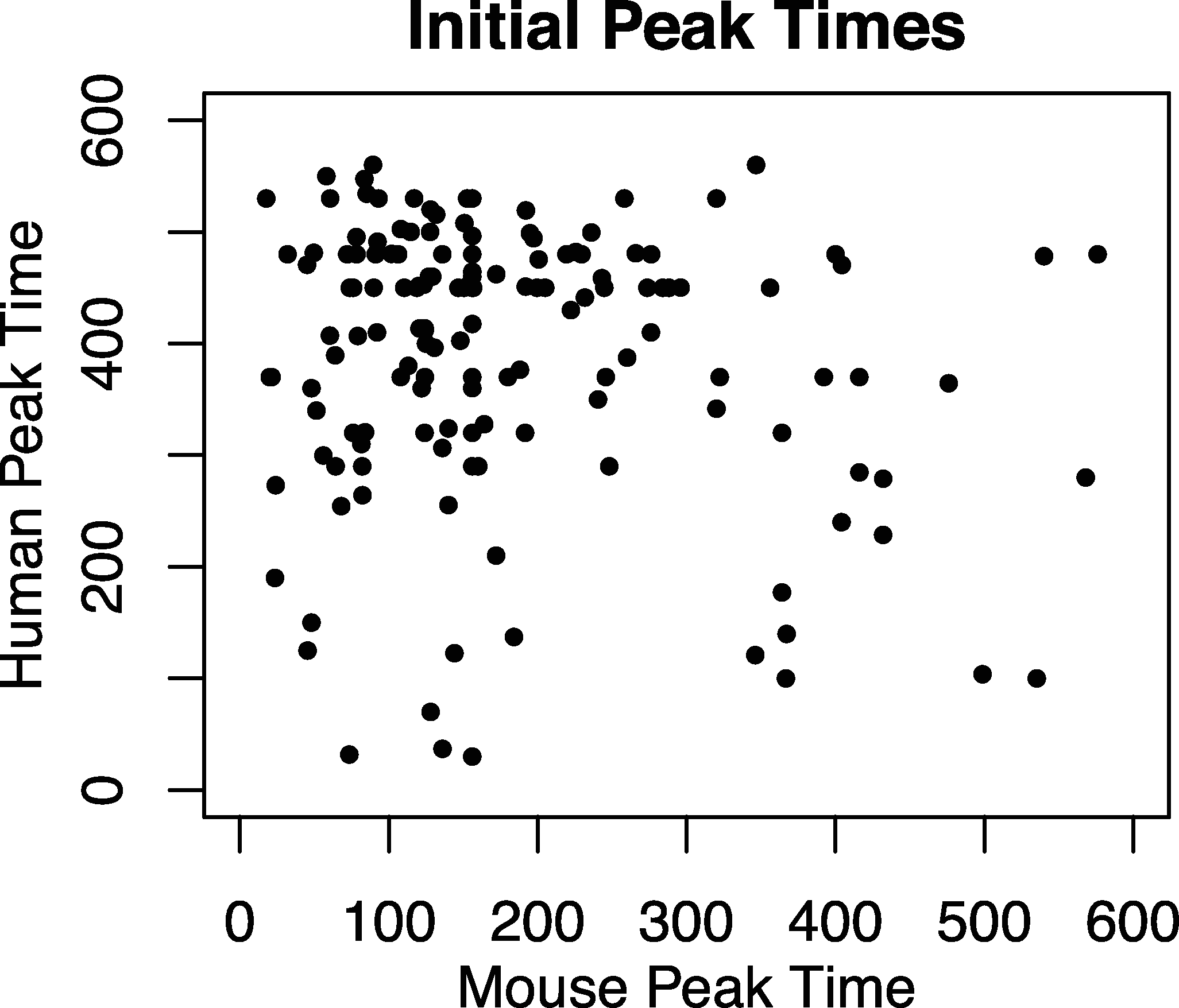

Supplement: S3 Fig — For all orthologs having at least one peak in both mouse and human cells, the time of the first (or only) peak is shown with the Mouse peak time on the x-axis and the Human peak time on the y-axis. (TIF) [file pcbi.1007543.s015.tif]

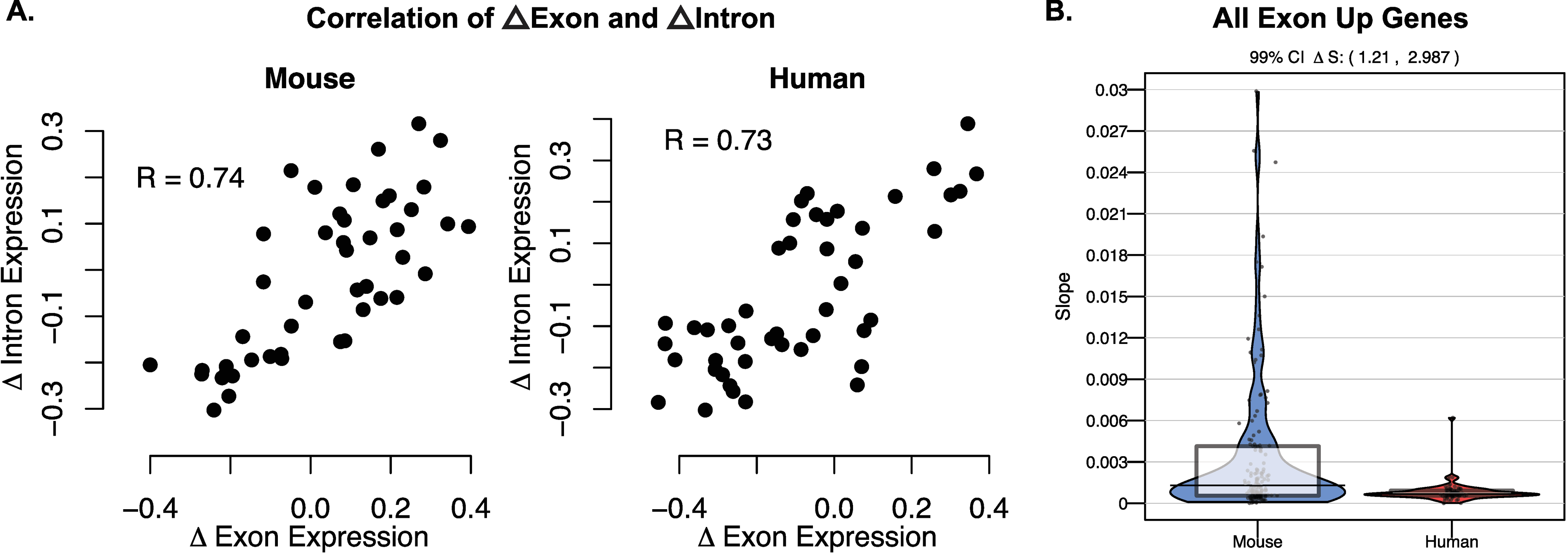

Supplement: S4 Fig — A) Treating the first and last ten time-points as a ‘condition’, the mean change in exon reads versus mean change in intron reads is shown for the human and mouse time courses. The high correlation indicates transcriptional activity is detectable. B) The intronic read slopes between mouse and human cells for genes that also were upregulated in either mouse or human exonic reads. (TIF) [file pcbi.1007543.s016.tif]

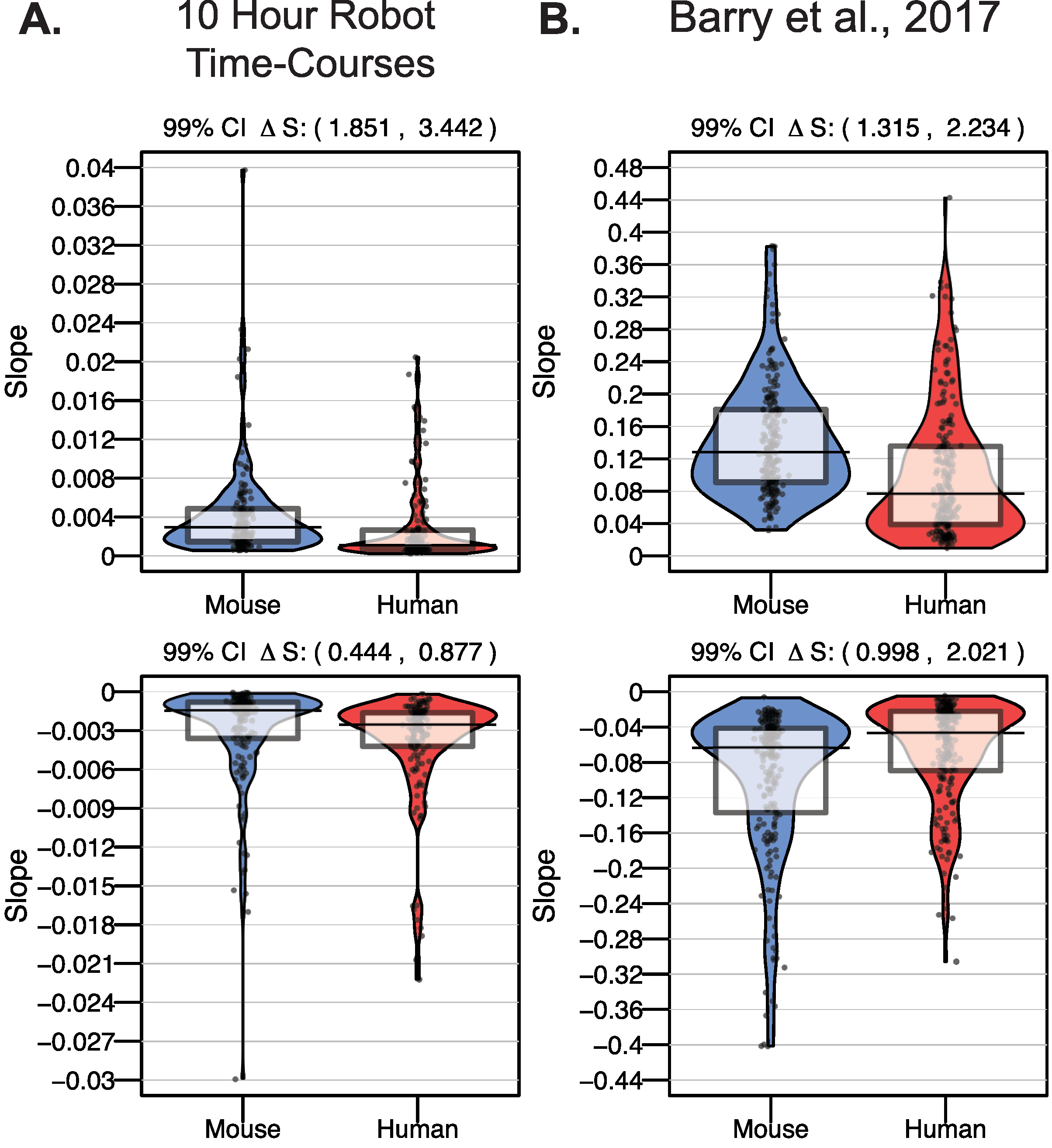

Supplement: S5 Fig — A) For all ortholog peak genes, the slopes of the up-trend (top) and down trend (bottom) are shown in a boxplot for the 10 hour robot time course. B) The same plots are shown for ortholog peaks in mouse and human time courses of three and six weeks, respectively, in Barry et al., 2017. (TIF) [file pcbi.1007543.s017.tif]
